# Mood disorders and suicides during coronavirus pandemic

**DOI:** 10.1192/j.eurpsy.2021.789

**Published:** 2021-08-13

**Authors:** K. Shah, C. Trivedi, H. Mekala

**Affiliations:** 1 Department Of Psychiatry, Griffin Memorial Hospital, Norman, United States of America; 2 Research, St Davids Healthcare, Austin, United States of America; 3 Psychiatry, Griffin Memorial Hospital, Norman, United States of America

**Keywords:** Covid, Suicide, mood disorder

## Abstract

**Introduction:**

The outbreak of COVID-19 has disrupted the lives of countless people worldwide. The pandemic has imposed a sense of uncertainty and anxiety, as the world could not predict or prepare for this crisis. It is important to study risk factors, including employment, marital status, and pre-existing medical or psychiatric conditions to effectively handle this pandemic’s mental health impact.

**Objectives:**

We aim to evaluate factors contributing to the suicides and mood disorders during the coronavirus pandemic.

**Methods:**

We examined MeSH terms “COVID-19” in the context of “Mood Disorders,” “Suicide,” “Suicidal Ideation,” “Assisted or Suicide, Attempted or Suicide,” “Risk Factors.” We identified eight case studies for the qualitative synthesis per the PRISMA guidelines, searching Medline, PubMed, PubMed Central, and PsychInfo databases until August 2020.

**Results:**

We identified that the population of all age groups and sex are at risk of stress and mental illness due to the pandemic. Several factors are attributed to the increased risk of mood disorders and suicide. Not having pre-existing psychiatric or medical condition is not a protective factor, since suicide was attempted or committed due to external factors such as economic and social.

**Conclusions:**

The pandemic has increased the risk of mood disorder and suicides in the population. Focus should be on the behavioral and psychological first aid to curb stress.

